# Medical and Philosophical Intelligence

**Published:** 1814-10

**Authors:** 


### MEDICAL AND PHILOSOPHICAL INTELLIGENCE.

Our readers will recollect the experiments of M. LECHENAULT on the deleterious effects of the juice known at Java by the name of *upas*, when introduced into wounds, as well as those of Messrs. DELILLE and MAJENDIE, which tend to prove that it is essentially on the spinal marrow that this poison acts.

Having been frequently witnesses of the frightful rapidity of its action, Messrs. Delille and Magendie were tempted to doubt that it could have been transported so quickly into the marrow by the tortuous and intricate way of the lymphatics; and they inquired if we ought not to admit, at least in certain cases, the absorbent faculty generally ascribed to the veins when we were not so well acquainted with all the ramifications of the lymphatic system. In order to come to some conclusion in this respect, they applied the upas to parts which adhered to the animal body by blood-vessels only: for example, they cut off all the mesentery adhering to the intestine of a goose, leaving only the arteries and veins; and after having placed the upas in the interior of this goose, they cut it, and tied both ends: nay, what appears still more conclusive, they cut a thigh, leaving the vein and artery only entire, and afterwards applied poison to the foot: finally, in order to remove even the objection of invisible lymphatic vessels, which might have belonged to the texture of these two blood-vessels, they removed a segment of both, after having supplied their place with quills, so that there was no longer any communication between the member and the animal, than by the blood which circulated from the one to the other. In all the experiments, convulsions and death came on as promptly as if the upas had been applied to the entire animal. Some, however. will still object, perhaps, that when the upas was introduced into the intestine, it might always be supposed that there remained some concealed lymphatic vessel; that, when it was applied to the foot, it was inserted into a wound, from which it could penetrate into the blood by open veins and arteries; and that this is by no means what is meant when we admit the venous absorption, for in that case we mean an action attributed to the veins in their natural state, and by their organic pores. What is still more remarkable in the experiments of Messrs. Magendie and Delille, is that the blood of an animal already poisoned, and ready to die, when transfused into the veins of another animal, does not kill the latter, and scarcely occasions to it any inconvenience.

M. Magendie has made another very interesting application of this action of certain substances, when introduced into the blood.

We know that an emetic injected into the veins of an animal makes it vomit in a few minutes, whilst it requires a whole hour when an emetic is swallowed to produce the same effect: and we instantly conclude, that this convulsive movement does not depend on the immediate action of this remedy on the coats of the stomach.

Observations

Observations made on the viscus itself, during the operation of vomiting, have led some physiologists still further. They perceived that the coats of the stomach underwent very little agitation; and hence they concluded also, that it is not in the irritation of these coats that the immediate cause resides of the expulsion of the contents of the stomach. Their opinions, however, were but feebly supported, and have almost fallen into oblivion since Lieutaud and Haller introduced one directly contrary.

M. Magendie, wishing to ascertain the truth, employed the convenient method of injections; and, having first made an opening in the abdomen, he ascertained by the touch, that during vomiting the stomach itself remains in a state of inertia, but that at every successive retching it is violently compressed by the contraction of the diaphragm and the muscles of the lower belly: besides, the long inspirations which precede every vomit, introduce a sufficiency of air into the stomach to prevent its capacity from diminishing, notwithstanding the quantity of matter which it ejects. If we open the abdomen wide enough to let out the stomach, the nauseæ continue; but they become impotent, because the muscles no longer compress the viscus: when we replace the stomach, the vomiting immediately begins. Compression is not sufficient of itself, however; for, if we compress with our hands a stomach displaced as above in a dog into whose veins no emetic has been injected, we can very well expel its contents without producing thereby a true vomiting, because there are neither nauseæ nor inspirations attending this kind of convulsion: but, if we pull the stomach instead of compressing it, and if we extend the pulling to the œsophagus, the nausea and all the other symptoms of vomiting appear, without there being any occasion for an emetic. Thus, vomiting would result from the compression exercised on the stomach by a convulsive contraction of the muscles which surround the belly; and this contraction itself may be excited by an irritation of the œsophagus.

It being of importance to know what muscles chiefly acted, what nerves put them in motion, and by what causes they were irritated, M. Magendie in the first place cut or removed the abdominal muscles, without much diminishing the activity of the vomiting: on the contrary, when we take from the diaphragm a great part of its strength by the section of the phrenic nerves, there are nothing but small retchings at long intervals, and the vomiting rarely takes place, notwithstanding the abdominal contractions. Thus, the part acted by the diaphragm in this compression is by far the greatest. When we thus destroy at once the action of the diaphragm and that of the muscles, the vomiting no longer takes place, even if we make the animal swallow substances eminently and promptly emetic, such as corrosive sublimate. Finally, M. Magendie entirely removed the stomach: he substituted for it a bladder, which he attached permanently to the base of the œsophagus, by making it communicate with this conduit by a solid tube; and after again sewing up the abdomen, he injected some emetic into the veins: the animal had nauseæ, made inspirations, and ejected a coloured liquid, (with which the bladder had been partly filled,) quite as well as it could have done, if, with a natural stomach, an emetic had been administered in the common way.

Thus, an emetic does not cause vomiting by irritating the fibres of the stomach, nor even the nerves, but by acting by means of absorption and circulation on the nervous system, and by exciting an action which is reflected specifically on the œsophagus and diaphragm, so as to make them exert various movements, among which there are some, the definitive result of which is the compression of the stomach: this does not prevent there being vomitings produced by the immediate irritation of the nerves of some of these parts, or by any given nervous irritation which would be propagated so as to affect the system nearly like an emetic.

It remains to M. Magendie to distinguish with more precision the part acted by the œsophagus and the diaphragm in the act of vomiting, and to examine the phenomena of this movement in birds and other animals which have no diaphragm.

To these experiments with antimony considered physiologically, M. Magendie added some others upon its medical or deleterious action; and he ascertained by many observations made upon human beings, and by several experiments upon animals, that the tartrite of this metal, taken in large doses, is of itself a deadly poison; but that almost always its first effect is a vomiting, which rejects the greater part before any mischief has been done: in this way many suicides are disappointed in their melancholy intentions.—*Phil. Mag.*

---

A case of *locked jaw* has lately occurred under the care of Dr. Phillips of Andover, in which the most decided good effects were supposed to be produced by the *Ol. Terebinth.* given as a clyster. The case will be published.

---

Dr. Babad, a physician at Roanne, lately read before the Medical Society at Paris, an account of the propriety of bleeding in intermittents. He commenced by stating the case of a plethoric young man, who had a quartan intermitting fever, which gave way, after the fifth accession of fever, to an emetic which was given on the third day, and a few doses of bark. Two weeks after he had a relapse of fever with the same type. At the fifth accession of fever, he had considerable pain, a strong full pulse, flushed face, his eyes prominent, pain and heaviness in the head; his ideas were confused, and his speech incoherent, with a strong feeling of suffocation. These symptoms determined Dr. Babad to bleed immediately, which produced syncope, and an involuntary motion. When he recovered from the syncope, the paroxysm had left him: bark was prescribed, but the fever did not return. The same man was attacked the following year with the intermittent, having analogous symptoms, but less intense, which bleeding immediately cured. M. Babad had also to congratulate himself on the happy termination of another case, from the use of the lancet. He concludes his cases with observing, that the symptoms which indicate bleeding in intermitting fevers,

 are, are, 1st, a sanguineous temperament, and plethoric habit; 2nd, a habitude of hæmorrhages and their suppression; 3rd, a fullness and hardness of the pulse; 4th, a sense of suffocation, difficulty of breathing, and strong beating of the heart; 5th, a tendency to apoplexy, which is characterised by the redness of the face, starting of the eyes, and pulsation of the temporal arteries.

---

Incontinence of urine is said to have been removed in a variety of cases by M. MONGERET, by the repeated application of Moxa on the sacrum. A report was made to the National Institute of seventeen cases, in which this remedy was completely successful.—*Gazette de Santé.*

---

M. MARSAN, professor of medicine at Padua, has drawn up a memoir upon a plant, more resembling the sugar-cane in its botanical characters, and in the quantity and quality of the sugar which it yields, than any hitherto discovered. It is a large gramineous plant from the South of Africa, described for the first time in 1775 by Peter Arduino, under the name of *holcus cafer*, and well characterised by its velvet down and globular seeds. It is now cultivating in various parts of Italy, Bavaria, and Hungary.

---

Indigenous coffee seems hitherto to have been less easily obtained in Europe than sugar: the torrefaction of many seeds and roots has been attempted with a view to procure substitutes, but the liquor produced by them had nothing of true coffee but its blackness and bitterness.

M. LEVRAT, a physician at Chatillon sur Chalaronne, thinks that the seed of the yellow water flag of our marshes (*iris pseudacorus*) is that which most approaches the coffee berry: after drying it by heat, and freeing it from the friable shell which envelopes it, it is then torrefied, and infused like coffee.

---

M. ORFILA, a young Spanish physician, has presented to the Institute an extensive work on poisons, considered with respect to medicine and medical jurisprudence. We have only perused the first volume, which treats of the poisons of mercury, arsenic, antimony, and copper. The author has detailed many experiments on the differences which the presence of various aliments acting as reagents, occasion in the operation of poisons, differences which may, in certain cases, disguise their properties, and prevent us from ascertaining them. He has pointed out all the precautions necessary for coroners, lawyers, and medical men, when the ends of justice are to be attained. He has particularly endeavoured, with the greatest care, to verify all the known methods of arresting the deleterious effects of these poisons, and to find new remedies where the old have failed. Thus, according to M. Orfila, the only antidote against corrosive sublimate is albumen or white of eggs diluted in water; and against verdigrese, common lump sugar, a result to which theory never would have led us.

4 M. MONTEGRE,

M. MONTEGRE, a physician at Paris, has made some curious observations on the habitudes of the *lumbrici* or earth-worms, and some new remarks on their anatomy. These animals are hermaphrodites, each being productive of young: nevertheless, there is no copula, or this seems to take place without any intromission of parts, and merely by the excitement of the movements necessary for fecundation. This takes place chiefly in June and July. The worms unite by means of a swelling at the anterior part of their body, and by which they adhere firmly to each other. The young worms first show themselves in white organs placed in front, on both sides of the stomach, and slide between the intestines and external muscles along a reservoir situated in the thick part of the tail, where they are found full of life. The *lumbrici* exhibited nothing to our observer which could induce him to ascribe to them the faculty of being affected by light or sound; but he was convinced that they did not confine themselves to the earth alone, for he found in their intestines the remains of animals and plants.—*Phil. Mag.*

---

*New Acid.*—GAY-LUSSAC has finished a very laborious and complete investigation of the properties of *iodine.* During his experiments he discovered that chlorine has the property of combining in two proportions with oxygen, and of forming two acids, which he calls the *chloric* and *chlorous* acids. The euchlorine of Davy is Gay-Lussac's chlorous acid; but it would seem that the chloric acid is the more curious and important compound.—*Dr. Thomson's Annals.*

---

*Agent for the Vaccine Disease.*—The government of the United States have authorized the appointment of an agent to preserve genuine vaccine fluid and furnish it to the citizens of the U. S. To facilitate his communications they have allowed him the immunity of the post-office. Dr. James Smith, of Baltimore, has been appointed agent, and has issued proposals for supplying the fluid, and determining, by inspection of the scab, whether the disease has been genuine.—*New England Journal.*

---

The practice of the London Infirmary for Diseases of the Eye, in Charter-house Square, under the medical superintendance of Dr. Farre and Messrs. Travers and Laurence, is open to students on the following terms:—For three months, five guineas; six months, eight ditto; as perpetual pupil, ten ditto.

---

*Theatre of Anatomy, Windmill-street.*—Mr. BRODIE will begin his Lectures on the Theory and Practice of Surgery, on Wednesday the 5th of October, at seven in the evening.

---

Mr. CARPUE will commence his usual Course of Lectures on Anatomy, the 1st of this present month.

---

Dr. SQUIRE will, on Tuesday the 4th of October, begin a Course of Lectures on the Theory and Practice of Midwifery, and the Diseases of Women and Children.

Dr.

Dr. Ramsbotham will begin his Lectures on Midwifery, including the Diseases of Women and Children, on Thursday, October 6th, at six in the evening, at his house, No. 9, Old Jewry.

---

Dr. Davis, Fellow of the Royal College of Physicians in Edinburgh, Licentiate of the London College, and Physician Man-midwife in ordinary to the Queen's Lying-in Hospital, will commence his first winter Course of Lectures on the Theory and Practice of Midwifery, and on the Diseases of Women and Children, on Wednesday the 5th of October, at a quarter past ten o'clock in the forenoon.

---

Mr. T. J. Pettigrew, F.L.S. will commence a Course of Lectures on Human Anatomy and Physiology, on Friday the 28th of October. The Course, to be comprised in eighteen Lectures, will be delivered on Wednesday and Friday evenings, at half past eight o'clock precisely. The introductory Lecture will consist of a general View of the Animal Structure.

---

*Hospitals for the Small-pox, for Inoculation, and for Vaccination, at Pancras, Middlesex.*—The following notice is given on a board attached to the premises: "Poor persons, of all ages, in the small-pox, are admitted any day and hour; and for inoculation, any morning from nine till eleven o'clock. Mothers and nurses may stay with children under five years of age, paying for their board. To out-patients, vaccination is giving daily from ten till twelve o'clock, Sunday excepted.

